# Model-independent fluxome profiling from ^2^H and ^13^C experiments for metabolic variant discrimination

**DOI:** 10.1186/gb-2004-5-12-r99

**Published:** 2004-11-16

**Authors:** Nicola Zamboni, Uwe Sauer

**Affiliations:** 1 Institute of Biotechnology, ETH Zürich, CH-8093 Zürich, Switzerland

## Abstract

We introduce a conceptually novel method for intracellular fluxome profiling from unsupervised statistical analysis of stable isotope labeling. Without *a priori *knowledge on the metabolic system, we identified characteristic flux fingerprints in 10 *Bacillus subtilis *mutants from 132 ^2^H and ^13^C tracer experiments. Beyond variant discrimination, independent component analysis automatically mapped several fingerprints to their metabolic determinants. The approach is flexible and paves the way to large-scale fluxome profiling of any biological system and condition.

## Abstract 2

A novel method for intracellular fluxome profiling that does not require *a priori *knowledge of the metabolic system allowed the identification of characteristic flux fingerprints in 10 *Bacillus *mutants from 132 ^2^H and ^13^C tracers experiments.

## Background

Genome-wide analyses of cellular mRNA, protein or metabolite complements have become workhorses in biological research that produce unprecedented amounts of data on cellular network composition. In contrast to such compositional information, molecular fluxes through intact metabolic networks link genes and proteins to higher-level functions that result from biochemical and regulatory interactions between the components [1]. As such, quantitative knowledge of *in vivo *molecular fluxes is highly relevant to functional genomics, metabolic engineering and systems biology [2,3]. Intracellular fluxes, or *in vivo *reaction rates, can be assessed by methods of metabolic flux analysis that are based on stable isotopic tracer experiments [4,5], which have successfully unraveled novel biochemical pathways [6,7] and gene functions [8,9]. The presently tedious and limited methodologies, however, hamper broader application to a large range of environmental conditions, isotopic tracers and higher biological systems [4].

We set out to overcome a principal bottleneck in metabolism-wide flux (fluxome [10]) analysis: the requirement for mathematical frameworks to interpret the isotopic tracer data from nuclear magnetic resonance (NMR) or mass spectrometric (MS) analyses within a detailed metabolic model [4,5]. Constructing such models requires *a priori *knowledge on possible distributions of the tracer used within the network, and, more importantly, extensive labeling and physiological data to resolve all fluxes within a given model. The lack of such structural knowledge on metabolic pathways and the technical difficulty of acquiring sufficient data hamper studies of metabolism, in particular in higher cells with complex nutrient requirements and for exotic tracer molecules. Hence, fluxome analysis is largely restricted to few ^13^C-labeled carbon sources in microbes or plants cultivated in minimal medium [7,11-16].

Here we discriminate mutants/conditions and assess their metabolic impact directly from 'raw' mass-isotope data by unsupervised multivariate statistics without *a priori *knowledge of the biochemical reaction network. To illustrate the applicability of this conceptually novel profiling method, we focused on the reactions of central metabolism in the model bacterium *Bacillus subtilis*, for which detailed flux data were available to validate the results [9,11,14].

## Results

### ^2^H and ^13^C tracer experiments

**Figure 1:**
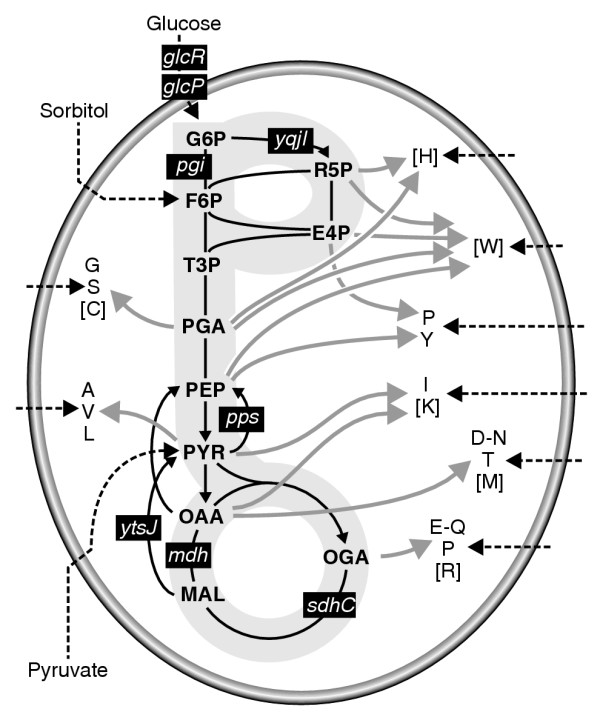
Simplified biochemical reaction network of *Bacillus subtilis *central carbon metabolism. Gray arrows outline the biosynthesis of precursor amino acids that are indicated by their one-letter code. Amino acids in square brackets were not detected. Black dashed arrows illustrate the uptake of substrates. Black boxes highlight pathways or reactions that are affected in the mutants used (see also Table 1). G6P, glucose 6-phosphate; F6P, fructose 6-phosphate; T3P, triose phosphate; PGA, phosphoglycerate; PEP, phosphoenolpyruvate; PYR, pyruvate; OAA, oxaloacetic acid; MAL, malic acid; OGA, 2-oxoglutarate.

Environmental and genetic modifications were used to perturb intracellular metabolic activities in *B. subtilis*. In particular, we chose 10 knockout mutants [17] that were affected in metabolic genes or transcriptional regulators linked to central metabolism (Table 1 and Figure 1). These mutants were grown in 1-ml batch cultures [18] with six combinations of the carbon sources [U-^13^C] or [U-^2^H]glucose, [U-^13^C]sorbitol or [3-^13^C]pyruvate and the nitrogen sources ammonium or casein amino acids (CAA). As a proof of concept, we detected the isotopic labeling patterns in proteinogenic amino acids by gas chromatography MS (GC-MS), which provides direct access to several metabolic nodes in the network [6,7,19] (Figure 1). The raw mass isotope data of all mutants under each of the six experimental conditions are given in Additional data file 2.

**Figure 2:**
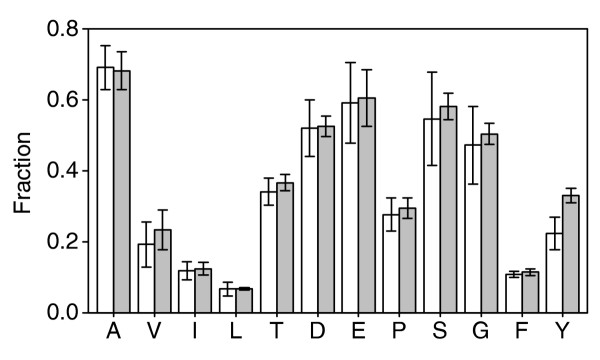
Fraction of amino acids that were synthesized *de novo *from [U-^13^C]glucose (white bars) and sorbitol (gray bars) in batch experiments supplemented with 0.5 g/l casein hydrolysate. Amino acids are given in the one-letter code.

In media supplemented with amino acids, cell protein was only partly synthesized from the isotopically labeled substrate. In such cases, current flux-analysis methods such as isotopomer balancing or flux ratio analysis are not applicable [4,5] because they do not account for variations in the labeling patterns due to amino-acid uptake and catabolism. Practically, we tackled here a worst-case scenario: growth in a medium enriched with unlabeled amino acids and profiling of the labeling pattern from tracers in the proteinogenic amino acids, which may potentially originate entirely from the medium. Nevertheless, a sufficiently high fraction of all analyzed amino acids was synthesized *de novo *from the labeled substrates to obtain relevant MS signals, indicating that information on pathway activities was recorded in the labeling patterns (Figure 2). To capture the impact of genetic or environmental modifications, we analyzed the 260-330 raw mass isotope data points for each mutant and condition. This is essentially a table of mass-distribution vectors for all detected amino-acid fragments upon correction for naturally occurring stable isotopes, that is, the list of the relative frequencies of all possible isotope isomers for each detected analyte.

### Identification of metabolic determinants for altered flux profiles

**Figure 3:**
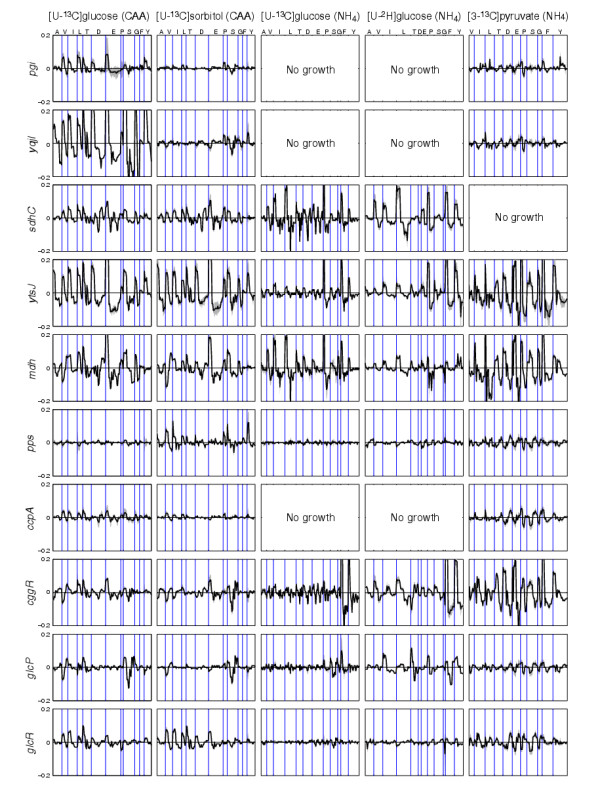
Comparison of labeling profiles in amino acids of *B. subtilis *mutants that were normalized by subtraction with the wild-type values obtained under the same condition, as obtained from five different medium compositions. The line deviates above (or below) the null line when an amino acid (represented by their one letter code at the top of the first panel) mass is more (or less) abundant in the mutant than in the parent. For each amino acid, the available data points are in the order of their total mass fragment. Gray areas represent the deviation of the normalized values, based on duplicate analyses of mutant and wild type. To reduce the dimension of the data for visual comparison, we excluded those values that, on average, accounted for less than 5% of the fragment pool in all mutants under a given condition.

**Figure 4:**
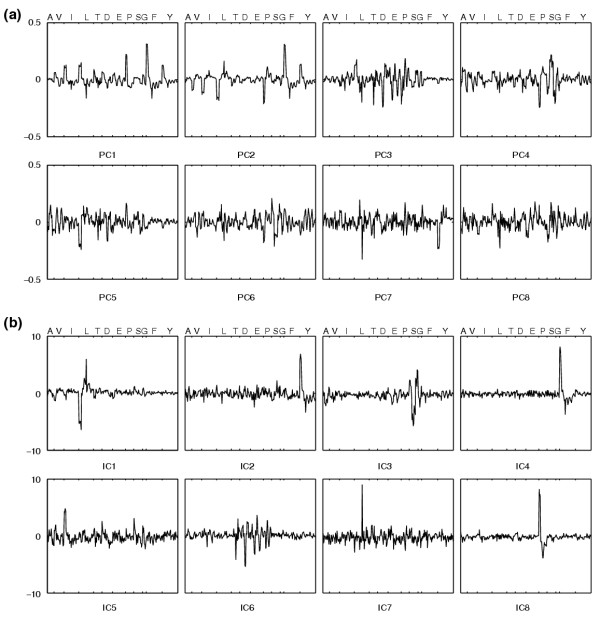
Weights of input variables. Weights of input variables in the first eight components obtained by **(a) **PCA and **(b) **ICA from the corrected MS data of the [U-^13^C]glucose experiment with ammonium.

For the visualization of metabolic effects, the corrected MS signals of the wild type were subtracted from those of the mutants (Figures 3 and 4). Some mutations, such as *pps*, were silent under the conditions tested and exhibited only noise in the wild-type-normalized data. In other mutants, characteristic profiles of strongly affected amino acids were readily apparent. One example was the almost identical signature of serine (S) fragments in the profiles of the *glcP *and *cggR *mutants during growth on sorbitol with CAA; that is, high fractions of masses *m*_0 _and *m*_3 _and low fractions of *m*_1 _and *m*_2 _(where the subscripts denote the number of ^13^C atoms in each amino-acid fragment). While the S signature of the *mdh *mutant on sorbitol with CAA was also distinct, it was different from that in the above two mutants with low *m*_1, _*m*_2, _and *m*_3 _fractions (Figure 3). These characteristic labeling profiles are biochemically very informative and may be linked to precise metabolic causes. For the above examples, the high fraction of uncleaved serine molecules with intact C_3 _backbones (that is, *m*_0 _and *m*_3_) in *glcP *and *cggR *is evidence of a lower exchange with the glycine pool, when compared with the wild type [19,20]. In the *mdh *mutant, the high fraction of uncleaved but unlabeled S (*m*_0_) reveals high incorporation of unlabeled serine from the CAA supplement, and thus low *de novo *biosynthesis from ^13^C-labeled sorbitol.

As well as consistency with the data in the literature, the analysis also revealed new information on pathway activity and regulation that was not previously accessible. One example is the pronounced signatures of the *sdhC *mutant on glucose and sorbitol. Because the *sdhC *mutation disrupts the tricarboxylic acid (TCA) cycle, the wild-type flux through the cycle must be similar on these substrates, both with and without CAA (Figure 3). The *sdhC *signatures of the TCA cycle-derived amino acids aspartate (D) and glutamate (E) were also present in the CAA profiles of the other TCA cycle mutant *mdh*. Their absence on ammonium indicates activity of the malic enzyme-based pyruvate bypass [11] in the *mdh *mutant.

While such a level of detailed biochemical insight is possible, it requires considerable expertise and time to retrieve. Alternatively, metabolic impacts in new mutants can be identified by comparison of the mass fingerprints in mutants with known metabolic lesions. During growth on sorbitol and pyruvate in minimal media but not with CAA, the CggR repressor of the glycolytic *gapA *operon, for example, appears to affect TCA cycle fluxes because the mutant profile matches those of the TCA cycle mutants *sdhC *and *mdh *(Figure 3). In contrast to glucose, sorbitol does not elicit catabolite repression; hence, comparison of sorbitol and glucose profiles can identify repression-dependent effects. Examples are the signatures of the oxaloacetate-derived amino acids isoleucine (I), threonine (T) and aspartate in the *cggR *profile that reveal, by the similarity to the *sdhC *and *mdh *mutants, a TCA cycle flux-promoting effect of CggR on sorbitol but not on glucose. This is consistent with the repression of *cggR *on glucose [21], and the TCA cycle effect is probably indirect, through the repression of glycolytic genes [22].

A significant extension beyond the canonical ^13^C-tracer methods is the applicability to any isotope, which broadens the observable metabolic processes. Here we used fully deuterated [U-^2^H]glucose that allows us to monitor dehydrogenase activities and water release. The ^2^H-label was present exclusively in the variable side chains, because the α-carbon hydrogen was lost in the transaminase reaction. Thus, glycine contains no label and the acidic aspartate and glutamate lose the label proximal to the carboxyl group as a result of exchange with water at the low pH during hydrolysis. The remaining amino acids provided a stable and informative ^2^H-pattern (see Additional data file 1). An illustrative example is the *cggR *mutant signatures for the pyruvate-derived amino acids valine (V), leucine (L) and, partially, alanine (A) (Figure 3) In all three cases, reduced *m*_2 _and increased *m*_0 _fractions revealed a double loss of ^2^H-label in their common precursor pyruvate at position C-3. This loss of ^2^H indicates increased exchange of ^2^H with water at the C-3 position of pyruvate (or any upstream triose), which is fully consistent with increased transcription of the glycolytic enolase in the *cggR *mutant on glucose [23] that could catalyze this exchange. As the enolase activity does not affect the carbon backbone, the corresponding patterns cannot be identified in ^13^C experiments

### Independent component analysis (ICA)

For large-scale profiling studies, automated mutant classification based on metabolic function without user supervision would be desirable. Initially, we used principal component analysis (PCA), which is often used for graphical representation of multidimensional variables from profiling experiments [24,25], as was recently described for pretreated (summed fractional labels) mass isotope data [26]. From the raw mass isotope data, the first two PCs discriminated, under most conditions, mutants with extreme labeling patterns (see Additional data file 1). The differences become smaller with increasing PCs, and only the initial three to four PCs allowed reliable discrimination. In the present data, PCA tended to discriminate extreme singular labeling patterns in few fragments or, more frequently, combinations of altered patterns in the fragments of many amino acids, as was expected from the variance maximization of PCA. Unfortunately, the resulting complex PCs are difficult to interpret metabolically, and thus are of limited biochemical relevance.

Consequently we used independent component analysis (ICA) for unsupervised, automatic recognition of conserved labeling patterns that are biochemically relevant. The underlying assumption is that these patterns result from the superposition of independent metabolic activities. Each activity causes a specific shift in the mass distributions of one or more intermediates. ICA seeks to separate the observed variables into non-gaussian components that are statistically as independent as possible [27]. Generally, ICA clearly discriminated mutants and conditions from the corrected (non-normalized) MS data (see Additional data file 1). While the weights in PCs were more broadly distributed among the input variables, ICs were dominated by fewer, sharper peaks (Figure 4).

**Figure 5:**
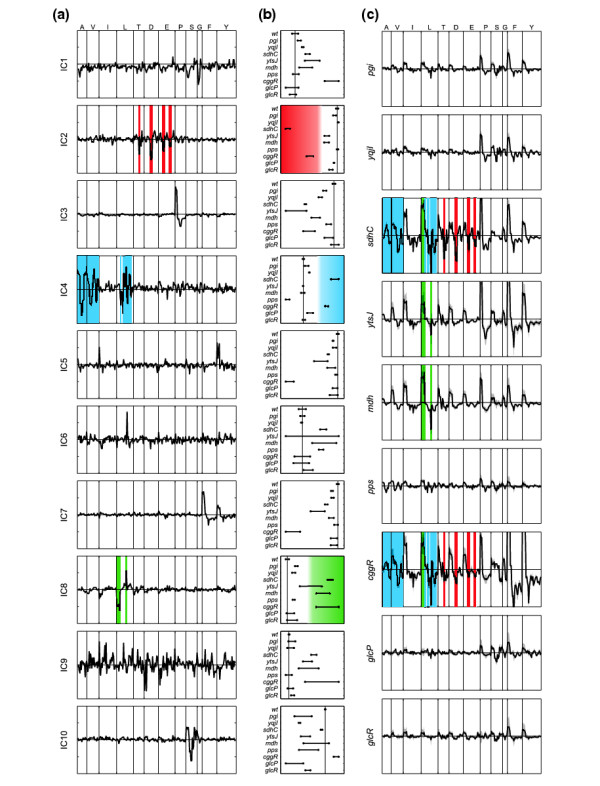
Fluxome profiling by independent component analysis of *B. subtilis *mutants grown on a 50:50 mixture of [U-^13^C]- and naturally labeled sorbitol with ammonium. **(a)** Weights of input variables (amino-acid mass-distribution vectors) in the mixing matrix of 10 ICs. **(b)** Projections (on *x*-axis) of samples on the IC shown in (a). The vertical line is drawn to intersect the average of the wild-type values. **(c)** Wild-type-normalized labeling profiles. Colors are used to highlight those aspects of the amino-acid profiles that were identified by ICA as relevant for the discrimination of the samples (b) along selected components.

For the particular example of the [U-^13^C]sorbitol with ammonium experiment, we explored the ICA results in more detail (Figure 5). The first, striking, observation was that the second IC contains the biochemically redundant signals of *m*_2 _T, *m*_2 _D, and *m*_1 _and *m*_3 _E (highlighted in red in Figure 5a) that arise from acetyl-CoA units in the TCA cycle [19]. This shows that ICA automatically provides insights into the biosynthetic linkage between amino acids with a resolution that eclipses visual comparison of the normalized signatures. For amino acids, this information was of course previously available, but statistical identification of biochemical relations could potentially also be obtained for less well-characterized compounds. Second, ICA often clustered biosynthetically related signals in the same component (Figure 5): IC7 grouped the similar signatures of phenylalanine (F) and tyrosine (Y) together; IC1 reports labeling shifts in glycine (G) and partially serine; and IC4 concentrated high weights in signals of the pyruvate derivatives alanine, valine and leucine (highlighted in blue in Figure 5). While isoleucine is also synthesized from pyruvate, it had only a marginal weight in IC4 because of interference from its second precursor oxaloacetate. Third, specific signatures of proline (P), leucine and serine are clearly recognized in IC3, IC8 (highlighted in green in Figure 5a), and IC10, respectively. These signatures reflect those previously identified in the normalized profiles (Figures 3 and 5c). Among the remaining components, IC5 and IC6 emphasize outliers in the *cggR *and *ytsJ *MS data, respectively, whereas the noisy IC9 profile indicates that the identified ICs in our small dataset approach a limit.

Akin to PCA, ICA allowed us to discriminate mutants from the corrected MS data (Figure 5b and Additional data file 1). On sorbitol, mutants such as *pgi*, *yqjI*, *pps, glcP *and *glcR *were mostly silent, and typically projected in proximity to the parent strain. In contrast to PCA, ICs classified the mutants on the basis of specific metabolic effects. In some cases (IC2 or IC4 in Figure 5b), the IC defined well-separated clusters of mutants, usually two groups, reflecting a binary (on-off) effect. In the majority of the components, however, the even distribution between the extremes reveals progressive metabolic responses (for example, IC3, IC7 or IC10). Overall, the ICs correlated favorably with the signatures of wild-type-normalized profiles (Figure 5 and Additional data file 1). Thus, ICA clearly outperformed PCA by its capacity for unsupervised recognition of metabolic responses and its ability to correlate biochemically redundant information in the data.

### Comparison of PCA and ICA with analytically determined flux ratios

**Figure 6:**
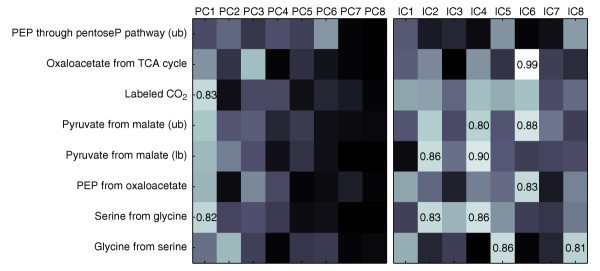
Correlation between analytically derived metabolic flux ratios (on the *y*-axis) [19] and the projections of the data on the first eight components obtained by PCA and ICA for the [U-^13^C]glucose experiment with ammonium. The brightness reflects the correlation coefficient, with black and white corresponding to values of 0 and 1, respectively. For coefficients higher than 0.8, the numerical value is reported. ub, upper bound; lb, lower bound.

For most experimental conditions tested, mathematical frameworks for numerical flux analysis such as isotopomer balancing or flux-ratio analysis [4,5] were not available. Only the [U-^13^C]glucose minimal medium experiments allowed a direct comparison of fluxome profiles with flux ratios. Therefore, we examined whether any of the statistically identified PCs and ICs was linearly correlated with eight analytically determined flux ratios [9,19] that were obtained from the same MS data (Figure 6). For PCs, the correlation coefficients decreased with increasing component number, and singular correlations could not be detected between individual PC-flux ratio pairs. Generally, the ICs were much better correlated with the flux ratios, for particular pairs with coefficients close to 0.90. This indicates that the identified ICs define signatures in the mass distribution of the analytes that bear high metabolic relevance, similarly to analytically derived flux ratios.

**Figure 7:**
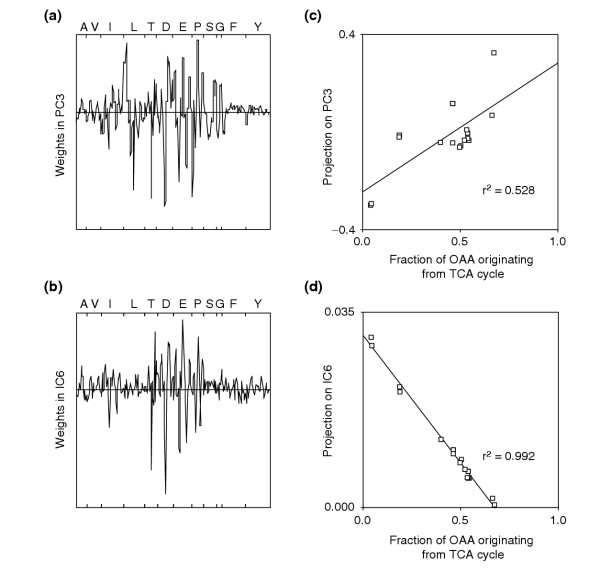
Weights of input variables in the component that is linked to TCA cycle activity, identified by either **(a) **PCA or **(b) **ICA from the [U-^13^C]glucose experiment with ammonium. In **(c) **and **(d)**, the projections of the mutant data on the component shown in (a) and (b), respectively, were plotted versus the analytically derived fraction of oxaloacetate (OAA) originating from TCA cycle [19]. The correlation coefficients are for linear fits.

Notably, IC6 was almost perfectly correlated with the flux ratio of oxaloacetate derived through the TCA cycle (Figure 6). This IC contained high weights in TCA-cycle-derived amino acids signals that are linked to the incorporation of C_2 _units from acetyl-CoA (Figure 4). As shown above, the projection of a data point on the axis defined by a component reflects the presence of the fluxome signature in its labeling patterns, and hence directly quantifies the occurrence of a particular metabolic activity. When plotting the projection versus the numerical values, the IC6-derived data exhibited a highly linear correlation, while the correlation coefficient was almost halved for PC3, the closest relative to IC6 (Figure 7). This confirms numerically the enhanced capacity of ICA to capture essential and independent information for a complex metabolic trait such as the TCA cycle activity. The extraordinarily high correlation coefficient of 0.99 demonstrates that IC6 represents very closely the analytically deduced TCA-cycle flux ratio. This is surprising because IC6 was statistically identified from 265 masses, whereas the flux ratio was calculated on the basis of a large body of biochemical background information [19,20].

## Discussion

For the example of central and amino-acid metabolism in *B. subtilis*, we show that fluxome profiling by multivariate statistics from mass isotopomer distribution analysis is meaningful for the discrimination of mutants or conditions on the basis of their metabolic behavior, and applicable to conditions that are inaccessible to previous flux analysis. In sharp contrast to metabolome concentration data [24,25], fluxome profiles contain functional information on the operation of fully assembled networks [1,4]. As shown here by ICA, this approach enables us to distill the essential signatures of independent metabolic activities, and supports the identification of the underlying biochemical causality. Because no model or *a priori *knowledge on the investigated system is required, the metabolic imprints of any tracer atom and molecule can be followed in virtually any biological system, including multicellular organisms in complex multisubstrate media.

Similarly, *a priori *knowledge of the number of ICs to be computed is not a prerequisite. As a matter of fact, the optimal number depends primarily on the labeling patterns and can hardly be estimated from the dataset dimensions. An underestimate will generally leave some relevant signatures unrecognized, whereas an overestimate will lead to an increased fraction of components reflecting measurement or biological noise. Although statistical significance can be assessed with duplicates, this becomes prohibitive with large datasets (that is, hundreds of mutants or analytes) or reduced availability of replicas. The bottleneck resides in the stochastic approach of most ICA algorithms, for which independent runs result in different ICs or ordering thereof. Instead, algorithmic and statistical reliability of the ICs can be evaluated by repeating the estimation several times either with randomly chosen initial guesses or by slightly varying the dataset (bootstrapping [28]), respectively, and then clustering all results to identify robust ICs [29].

Two factors directly affect the results that can be obtained by comparative fluxome profiling: the detected analytes and the choice of isotopic tracer. As well as polymer-based analytes such as the proteinogenic amino acids monitored here, fluxome profiles can be detected in any set of intra- or extracellular metabolites, thereby widening the observable metabolic processes The choice of tracer depends, to some extent, on the metabolic subsystem of interest. Uniformly labeled substrates provide a more global perspective because they allow assessment of the scrambling of any carbon backbone and, in the case of experiments performed in rich media, also allow quantification of the fraction of *de novo *biosynthesis from the tracer relative to the uptake of a medium component. Similarly, uniformly deuterated substrates or ^2^H_2_O are valuable for simultaneously capturing a wide number of ICs that are affected by the release, binding and exchange of water or protons. Substrates that are labeled at specific positions, in contrast, enable deeper interrogation of particular sub-networks, for example, [1-^13^C]hexoses for the initial catabolic reactions [8,19] or [1-^13^C]aspartate to assess urea cycle activity.

The results also revealed new biological information on pathway activity, function or regulation. First, both glycolysis and the pentose phosphate pathway actively catabolized glucose in the presence of CAA, because the *pgi *and *yqjI *mutant signatures were different from the wild type and from each other. On sorbitol, in contrast, the same mutants were very similar to the wild type, suggesting that both reactions are only marginally involved in catabolism of this sugar. Second, the Krebs cycle flux was similar on glucose and sorbitol (with and without CAA), as deduced from the similarly pronounced signatures of the *sdhC *mutant. Third, absence of the *sdhC *signatures in the Krebs cycle-derived amino acids aspartate and glutamate of the *mdh *mutant when grown with ammonium (but not CAA) indicates activity of the malic enzyme-based pyruvate bypass [30]. Fourth, activity of the NADP-dependent malic enzyme appears to be independent of catabolite repression because pronounced signatures of the *ytsJ *mutant were seen on all substrates. The gluconeogenic phosphoenolpyruvate synthetase Pps, in contrast, was inactive in the presence of the repressing glucose but active on pyruvate or sorbitol. Fifth, as discussed above the data reveal a Krebs cycle-promoting effect of the repressor CggR on sorbitol but not on glucose, most likely through the repression of glycolytic genes [22].

The comparative fluxome profiling presented here complements traditional flux analysis because it enables potentially rapid and automated identification of relevant mutants or conditions from large-scale datasets, for example from entire mutant libraries. The approach is quantitative in terms of the relative difference between variants, but qualitative with respect to the *in vivo *flux. Interesting variants are then subjected to deeper interrogation of the specific metabolic phenomenon identified. Besides mere data mining, fluxome profiling also has the potential to identify complex functional traits in higher cells where current flux methods fail, and possibly even identify the underlying biochemical mechanism of discriminant mass isotope signatures.

## Materials and methods

### Strains and growth conditions

Wild-type *B. subtilis *168 (*trpC2*) [31] and knockout mutants containing an antibiotic marker in single genes [17] were grown in M9 minimal medium [9] at pH 7.0 with 50 mg tryptophan. Six different combinations of ^2^H- or ^13^C-labeled isotopic tracers (3 g/l) and nitrogen sources were used: (i + ii) uniformly ^13^C-labeled [U-^13^C]glucose with either 0.5 g/l CAA (Sigma) or 1 g/l NH_4_Cl; (iii + iv) [U-^13^C]sorbitol with either 0.5 g/l CAA or 1 g/l NH_4_Cl; (v) [U-^2^H]glucose ([1,2,3,4,5,6,6-^2^H]glucose) with 1 g/l NH_4_Cl; and (vi) [3-^13^C]pyruvate with 1 g/l NH_4_Cl and twofold higher concentrations of phosphate to ensure pH buffering. [U-^13^C]glucose (Martek Biosciences), [U-^13^C]sorbitol (Omicron Biochemicals), and [1,2,3,4,5,6,6-^2^H]glucose (Euriso-Top) were supplemented as 50:50 mixtures of labeled and unlabeled isotopomers. Pyruvate was supplied entirely as the [3-^13^C] isotopomer (Euriso-Top).

Aerobic batch cultures were grown in silicone-covered, deep-well microtiter plates at 37°C and 300 rpm in a 5-cm orbital shaker [18]. Frozen stocks were used to inoculate 1 ml LB medium with selective antibiotics. After 10 h of incubation, 10 μl were used to inoculate 1 ml M9 medium with 5 g/l glucose and selective antibiotics, incubated for 12 h, and 10 μl of these precultures were used to inoculate 1.2 ml of M9 medium with isotopic tracers. Cultures were harvested upon entry into stationary phase (assessed by visual evaluation). Because the length of batch growth varied, cultures with CAA, with NH_4_Cl, and with pyruvate were harvested after 10, 14 and 24 h, respectively. Labeling patterns in the analyzed proteinogenic amino acids are rather stable [10,19]; hence differences of a few hours in growth phase at harvest were irrelevant. This was also confirmed in separate (data not shown) and duplicate experiments for each combination of strain and medium that was independently started from culture stocks.

### GC-MS analysis and data preprocessing

Cell harvest, protein hydrolysis and GC-MS analysis of amino acids were done exactly as described before [19,32]. Amino-acid mass distributions were derived from the spectra after correction for the natural abundance of stable isotopes [19]. Since amino acids are fragmented during electron impact ionization in the MS, we obtained three to five fragments with partially redundant information for each amino acid. For each fragment, a normalized vector *m*_0_, *m*_1_, ..., *m*_n_, expresses the fraction of molecules that are labeled at 0,1, ...,*n *positions, depending on the total number *n *of carbon or hydrogen atoms present. Considering all corrected fragment vectors obtained per sample, a complete dataset typically consisted of about 260 and 330 single mass values from ^13^C and ^2^H experiments, respectively, depending on the quality of the MS measurement.

### Multivariate data analysis

To obtain a new representation of the multivariate MS data and to make their essential structure accessible, we applied PCA to the corrected fragment vectors. This approach projects the input variables in an orthogonal space that is spanned by the PCs. Among the infinite number of possibilities, each successive PC is selected to maximize the variance of the projected data and to be orthonormal to the previous ones [33]. Consequently, PCA concentrates the maximum and nonredundant information of the entire dataset in the minimal number of dimensions, and thus is best suited for data compression [27]. The computation was performed with Matlab (The Mathworks) using the *princomp *function of the Statistics toolbox 4.0. No input vectors were eliminated from the dataset to filter outliers in PCA, because this operation affected only PCs with higher order but only marginally PC1 and PC2.

To reveal hidden information in the labeling patterns, the corrected MS vectors were subjected to ICA [27], which is frequently used in the neurosciences [34,35] and in gene-expression studies [36,37]. For ICA, we assume that independent metabolic processes such as reactions or pathways produce characteristic fingerprints in the labeling pattern. These metabolic fingerprints are defined by *m *fundamental components *S *= (*s*_1_, ..., *s*_*m*_)^T^, each of which is represented by a vector of *p *MS-signals. We assumed that the experimental data *X *= (*x*_1_, ..., *x*_n_)^T^, with *n *vectors of *p *corrected MS signals for each mutant/condition, result from a linear combination of the *m *fundamental processes, given by *x*_*i *_= *a*_*i*1_*s*_1 _+...+ *a*_*i*m_*s*_m_. In matrix notation, this leads to *X*_*p*__×__*n *_= *A*_*p*__×__*m*_*S*_*m*__×__*n*_, with *A *as the mixing or loading matrix. ICA seeks to estimate the unknown terms *A *and *S *from the observed values *X *but has different objectives from PCA. Briefly, ICA identifies statistically ICs by selecting those with maximum non-gaussianity [27]. Hence, ICs are nonlinearly decorrelated and assumed to have non-gaussian distributions. Because of the central limit theorem, which states that the sum of non-gaussian random variables is closer to gaussianity than the original ones, ICs are identified by selecting the linear combinations of the observed variables that have maximum non-gaussianity [27]. In particular, we used the publicly available FastICA 2.1 algorithm [38] to estimate the number of components that were equal to the number of strains in the dataset, excluding duplicates. The data dimension was not reduced (by PCA) before IC computation.

## Additional data files

The following additional data is available with the online version of this paper. Additional data file 1 contains three figures (Additional Figure 1 shows the mass distribution in the ^2^H experiment; Additional Figure 2 shows mutant discrimination by PCA (less relevant than by ICA); Additional Figure 3 is a complete representation of the 660 ICs (10 ICs in 6 experiments for 11 strains). All the raw data is contained in six Excel tables in Additional data file 2.

## Supplementary Material

Additional data file 1 Three additional figures (Additional Figure 1 shows the mass distribution in the ^2^H experiment; Additional Figure 2 shows mutant discrimination by PCA (less relevant than by ICA); Additional Figure 3 is a complete representation of the 660 ICs (10 ICs in 6 experiments for 11 strains) Click here for additional data file

Additional data file 2 All the raw data contained in six Excel tables Click here for additional data file

## Figures and Tables

**Table 1:** *B. subtilis *strains used

Strain	Description of deleted gene
Wild-type	168 *trpC2*
*pgi*	P-glucoisomerase
*yqjI*	6-P-gluconate dehydrogenase
*sdhC*	Succinyl-CoA dehydrogenase component
*ytsJ*	Malic enzyme
*mdh*	Malate dehydrogenase
*pps*	PEP synthetase
*ccpA*	Main carbon catabolite repressor
*cggR*	Repressor of the *gapA *operon
*glcP*	Hexose/H^+ ^symporter
*glcR*	Repressor of PTS system

Strains were provided by S. Aymerich (INRA, CNRS, Thiverval-Grignon, France) and K. Kobayashi (Nara Institute of Science and Technology, Nara, Japan) [17].
