# Supplementary material for: Model-independent fluxome profiling from 2H and 13C experiments for metabolic variant discrimination
Source: Genome Biol. 2004 Nov 16;5(12):R99. doi: 10.1186/gb-2004-5-12-r99 (PMC545802; doi:10.1186/gb-2004-5-12-r99)

# Supplementary FIGURES

**Supplemental Figure 1**  Mass distribution obtained from GC-MS analysis of proteninogenic amino acids in *B. subtilis* mutants grown on a 50:50 mixture of either [U-^13^C] or [U-^2^H]glucose and glucose labeled at natural abundance.


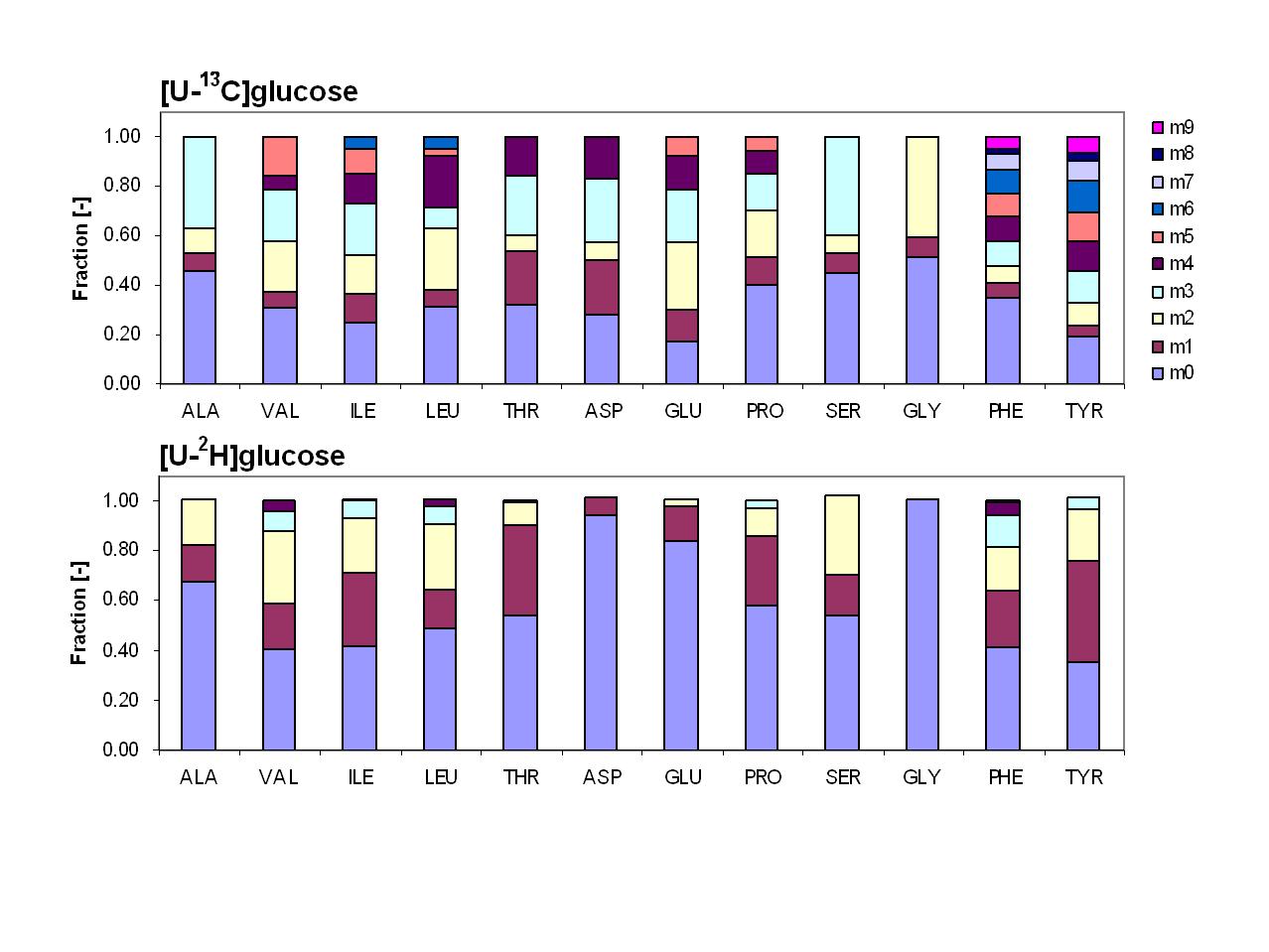


**Supplemental Figure 2** Mutant discrimination by projection into the planes spanned by the first two principal components of the mass distribution vectors of all detected amino acid fragments from different isotopic tracer experiments. The variance of each principal component relative to the entire data set is indicated on the axes. Discriminated mutants are denoted with the abbreviated name of the inactivated genes (compare Tab. 1), and the wild-type is highlighted by the open squares. Independent duplicates are connected with lines. The nitrogen source in each experiment is given in parenthesis. For each mutant and condition, the mass distribution vectors of proteinogenic amino acids were determined by GC-MS analysis.

**
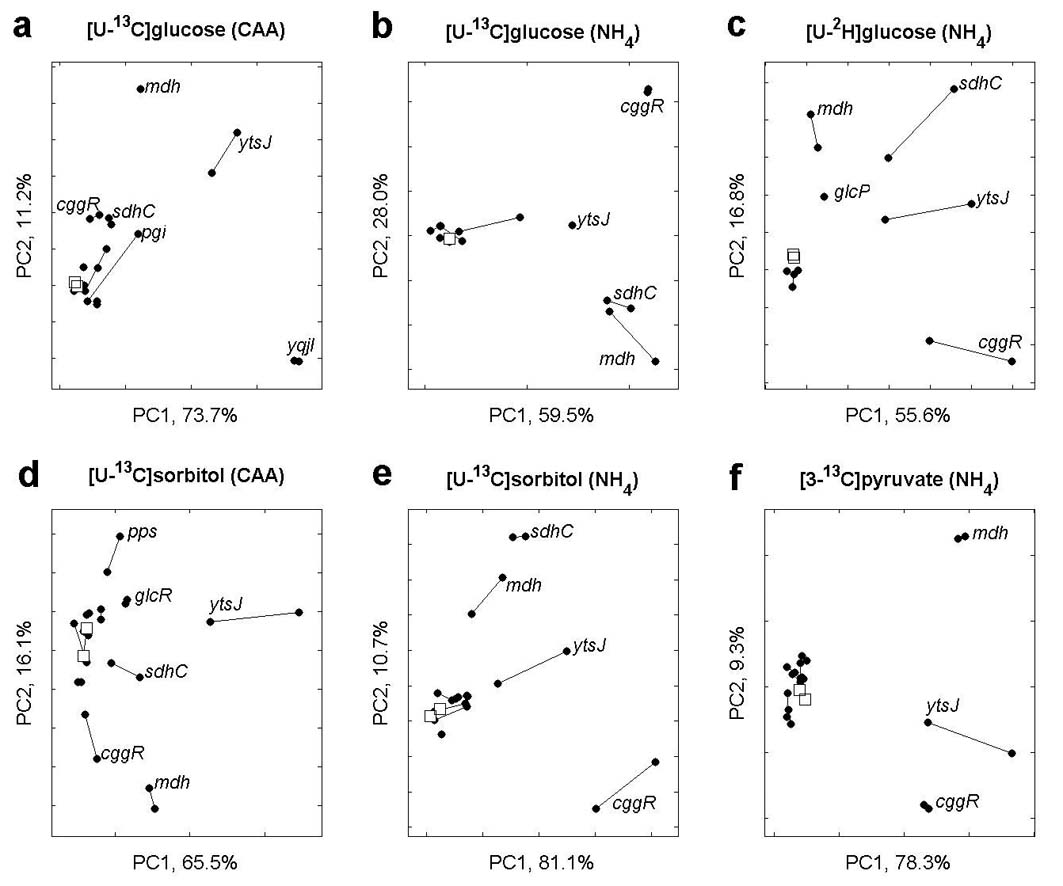
**

**Supplemental Figure 3**  Independent component analysis of corrected MS data for the six combinations of isotopic tracers and conditions tested. For each experiment (on the left), the projections (on *x*-axis) of samples on ten ICs are shown on a row. The vertical line is drawn to intersect the average of the wild-type values.

See next page!


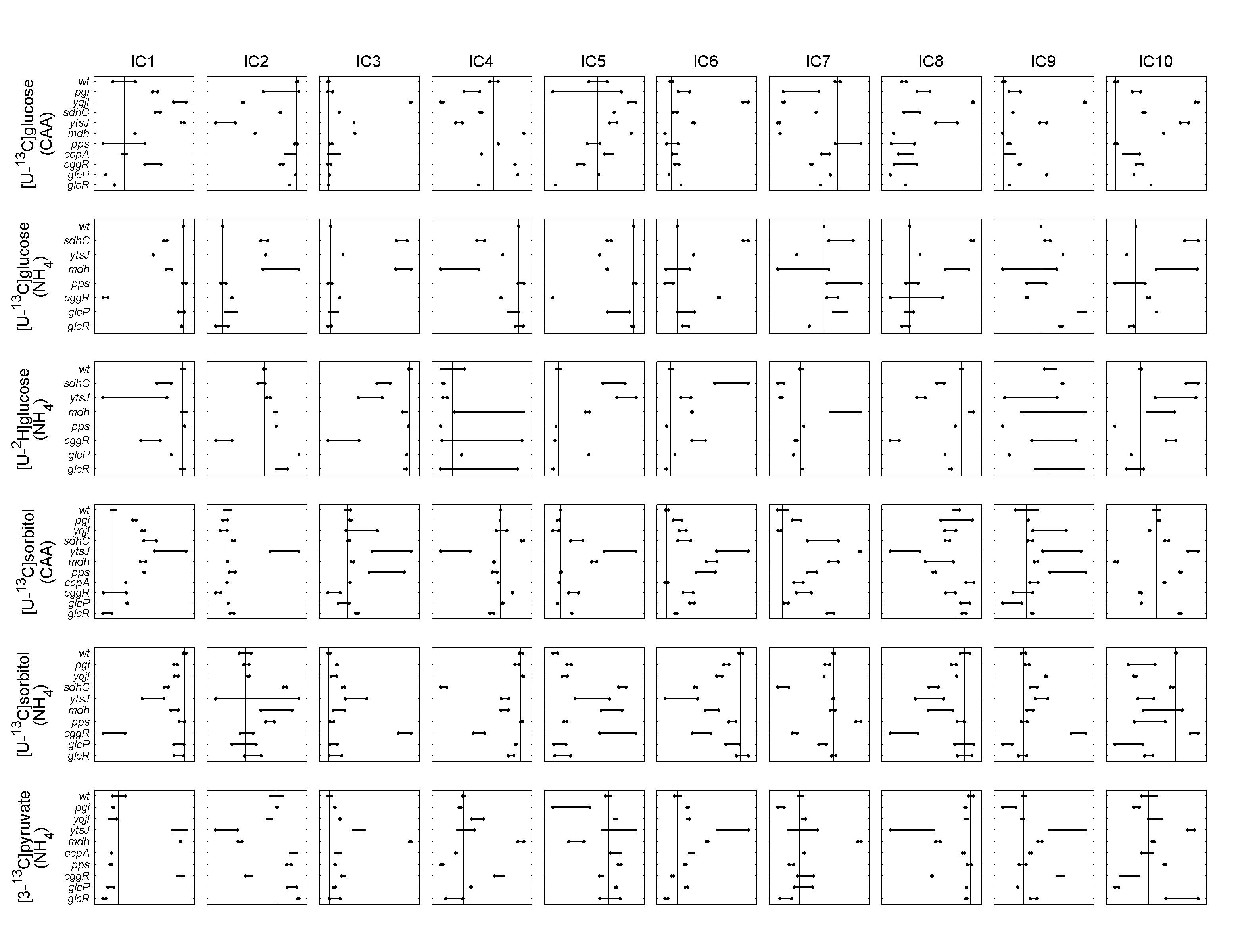

Supplement: Additional data file 1 — Three additional figures (Additional Figure 1 shows the mass distribution in the 2H experiment; Additional Figure 2 shows mutant discrimination by PCA (less relevant than by ICA); Additional Figure 3 is a complete representation of the 660 ICs (10 ICs in 6 experiments for 11 strains) [file gb-2004-5-12-r99-s1.doc]
